# Supplementary material for: Citizen attitudes towards the environment and association with perceived threats to the countryside: Evidence from countries in five European biogeographic zones
Source: PLoS One. 2024 Oct 10;19(10):e0311056. doi: 10.1371/journal.pone.0311056 (PMC11469601; doi:10.1371/journal.pone.0311056)
Supplement: S1 File — (DOCX) [file pone.0311056.s001.docx]

## **S1 File: Cross-national item-specific comparisons**

Item-specific relationships indicated that, while the attitudinal and perceived threat to the countryside measures had substantial commonality across the five countries, unique differences were also apparent for many of the items. Only perceived threat items 1 ‘conversion to urban land use’, 6 ‘increased number of visitors’ and 11 ‘changing diets’ had all country responses in agreement. The Swiss sample had two perceived threat items which differed from the UK and Swedish samples (items 2 ‘conversion of pasture or meadows to crop land’ and 8 ‘poor farming practices’). Within the Spanish sample, perceived threat item 2 ‘conversion of pasture or meadows to crop land’ was adjusted, as in the Swiss sample, but in addition, items 13 ‘farmers unable to make a living from the land’, 14 ‘lack of young farmers taking over farming’, and 15 ‘changes to the market prices of farm products’ were different. Items 14 and 15 in the Czech dataset also had a specific effect from the utilisation factor, but in this case the relationship was in a negative direction when compared with the Spanish sample. This specific effect (Item 8) occurred in both the Czech and Swiss samples. Items 3 ‘conversion of pasture or meadows to forest’, 5 ‘to many livestock causing damage to the land’, and 10 ‘effects of climate change’ were unique to the respondents in the Czech sample. Differences across countries appear relatively minor, but as more countries were included, the greater the discrepancy that occurred in the measures. It is therefore important to consider not just universal measures (total scores) and invariant factors when making model comparisons, but also country unique (specific) responses. A multiple indicators multiple causes (MIMIC) approach within a mediator and moderated model facilitated the exposure of these specific effects.
