# Supplementary material for: Citizen attitudes towards the environment and association with perceived threats to the countryside: Evidence from countries in five European biogeographic zones
Source: PLoS One. 2024 Oct 10;19(10):e0311056. doi: 10.1371/journal.pone.0311056 (PMC11469601; doi:10.1371/journal.pone.0311056)
Supplement: S1 Table — (DOCX) [file pone.0311056.s002.docx]

**S2 Table. Results of mediating effects.**

| **Country** | **Results** | **Modelling adjustment** |
| --- | --- | --- |
| ***UK*** | In the UK, higher environmental attitude inventory (EAI) scores on the preservation factor (preservationist attitudes) were associated with higher scores on perceived threats to the countryside (Table 6). EAI scores on the utilisation factor (utilitarian attitudes) were unrelated to perceived threat to the countryside. Environmental utilitarian attitude scores were higher for men, younger, and the less educated respondents. Environmental preservationist attitude scores were higher in women, older people, those who had spent longer in education, and area associated with more frequent visits to the countryside. No associations were observed between UK respondents’ residency (urban *versus* rural dwellers) and either utilitarian or preservationist attitudes. There were no significant differences in perceived threat to the countryside within the UK sample on any of the exogenous variables (age, the frequency of visiting the countryside, gender, education level or residency). UK respondents’ age (being older) (*P*=0.031), gender (being a woman) (*P*=0.044), education level (being more educated) (*P*=0.042) and more frequent visits to the countryside *P*=0.033) had an indirect positive association with perceived threat to the countryside through preservationist attitudes. | In the UK model (i.e., the baseline model) a number of item-specific effects occurred (3, 4, 7, 9), which were affected by the utilisation attitude and item 10, which was affected by preservationist factor. These relationships were then put in place across the countries. These items all had in common ‘proper use of land’ on the utilisation measure and on the perceived threat to the countryside items. The preservationist factor on item 10 of the perceived threat to the countryside factor related to climate change. Responses to items 3 and 4 indicated a more positive attitude to the conservation of land, while a higher score on the utilisation factor was related to viewing (a) bad behaviour on the land (item 7) and (b) chemical misuse as less of a threat (item 9). |
| ***Czechia*** | Higher environmental attitude inventory (EAI) scores on both the preservationist and utilitarian EAI factors were associated with higher perceived threat to the countryside in Czechia (Table 6). Utilitarian environmental attitudes were higher in women than men. There were no associations between any exogenous factors (age; gender; education; residence) and preservationist environmental attitudes. Perceived threat to the countryside increased with education level. There was no association between perceived threat to the countryside, age and the frequency with which people visited the countryside, or between gender or rural *versus* urban dwellers. In Czechia, gender (being a women) and had an indirect positive association with perceived threat to the countryside which was mediated by utilitarian environmental attitudes (*P*=0.004). | With the testable restriction in place, data for Czechia were added to the analysis. With data from Czechia some additional specific items came into play (utilisation factor: items 5, 8, 10, 14, 15, and with the preservationist factor item 3). Items 8, 10, 14 and 15, all had a negative association, indicating that a high score on utilisation was related to a low score on endorsing items relating to farming. Item 5 related to damage of land by too many livestock and this was more likely to be positively endorsed by those higher on utilisation. Conversion of land to woodland (item 3) was also related positively to the preservation factor. |
| ***Switzerland*** | In Switzerland, higher perceived threat to the countryside was associated with higher scores on both the preservationist and utilitarian environmental (EAI) factors (Table 6). Higher EAI scores on the environmental utilitarian factor were associated with being a woman, younger and educated to a higher level. Higher EAI scores on the preservationist factor were associated with more frequent visits to the countryside. There was no association between perceived threat to the countryside and age or the frequency with which people visited the countryside, or between gender, education level or rural *versus* urban dwellers. In Switzerland, age (being younger) was negatively associated with perceived threat to the countryside indirectly through utilitarian attitude (*P*=0.009). Gender (being a woman) was positively associated with perceived threat to the countryside indirectly through utilitarian attitude (*P*=0.007). More frequent visits to countryside (*P*=0.032) were positively associated with higher perceived threat to the countryside indirectly through preservationist attitudes. | In the Swiss sample, two additional items, over and above those already present from the baseline model, were specified (item 8 relating to the effect of the preservation factor on perceived threat to the countryside and item 2 on the utilisation effect on perceived threat to the countryside). Item 8 referred to poor farming practices and this was likely to be less strongly endorsed when scores on the preservationist factor were high. Where scores were higher on the utilisation factor, the conversion of pastures or meadows to crop land was more highly endorsed. |
| ***Sweden*** | Higher environmental attitude (EAI) scores on both the preservationist and utilitarian factors were associated with higher perceived threat to the countryside among those recruited in Sweden (Table 6). Higher EAI scores on the utilitarian factor were associated with being younger and higher scores on the preservationist factor were associated with being older. Perceived threat to the countryside was significantly higher in men and with increasing age. In Sweden, age (being younger) was indirectly and negatively associated with perceived threat to the countryside indirectly through the EAI utilitarian factor (*P*=0.010). Being older was positively associated with perceived threat to the countryside indirectly through the EAI preservationist factor (*P*=0.010). | In the Swedish sample only item 12 ‘changing demand for food’ needed an item-specific adjustment in relation to the utilisation factor in addition to those already introduced for the baseline model. |
| ***Spain*** | Higher environmental attitude (EAI) scores on both the preservationist and utilitarian factors were associated with higher perceived threat to the countryside among those recruited in Spain (Table 6). Higher EAI scores on the utilitarian factor were associated with being a woman, younger and less educated. Higher scores on the preservationist factor were associated with being a man and older. Perceived threat to the countryside increased with age. In the sample recruited in Spain, age (being younger) was negatively associated with perceived threat to the countryside indirectly though utilitarian attitudes (*P*=0.005). Age (being older) was positively associated with perceived threat to the countryside indirectly via preservationist attitudes (*P*=0.020). Gender (being a woman) was positively, indirectly, associated with perceived threat to the countryside via the EAI utilitarian factor (*P*=0.007) and being a man was indirectly and negatively associated with perceived threat to the countryside through preservationist attitudes (*P*=0.004). | Items 13, 14 and 15 were added as additional variables in the Spanish sample data. These effects came from the utilisation factor (a) farmers unable to make a living, (b) lack of young farmers taking over farming, and (c) changes to market prices for farm products. The latter two (items 14 and 15) had a positive relationship to having a higher score on utilisation, while item 13 had a negative relationship. The attitudinal factor relating to preservation was positively associated with item 2, which referred to the conversion of pastures or meadows to crop land. |
