# Supplementary material for: Citizen attitudes towards the environment and association with perceived threats to the countryside: Evidence from countries in five European biogeographic zones
Source: PLoS One. 2024 Oct 10;19(10):e0311056. doi: 10.1371/journal.pone.0311056 (PMC11469601; doi:10.1371/journal.pone.0311056)
Supplement: Attachment — Submitted filename: Response to ReviewerS Comments.docx [file pone.0311056.s003.docx]

**Authors’ Response to Reviewers**

**Response to Reviewer 1:**

**Comment 1:**

Reviewer #1: Based on the sample of five European countries in different geographical environments, this paper uses the structural model to survey the citizen attitudes towards the environment and association with perceived threats to the countryside, which is significant for promoting public support for environmental protection policies. However, reading this manuscript, some apparent problems exist and need further improvement. The comments are as follows:

1. The introduction needs to summarize and refine the innovation of your article better. Compared with existing research, how innovative is the article? This description needs to be stronger in the introduction.

**Authors’ response:**

Taking the reviewers advice, we have updated the cited literature and in doing so have strengthened the introduction section (especially lines 144-217). We have also developed the section at the end of the introduction and have pinpointed exactly how the research is novel (e.g. topical; large sample across different countries and geographical area; employment of a well-validated attitude scale; provides implications for policy on environmental protection) (**lines 200-217**).

**lines 200-217**

*To the authors knowledge, previous research has not considered the relationship between environmental attitudes and perceived environmental threats to the countryside, nor has there been comparative analysis of the perceptions and attitudes of large representative samples cross-nationally in countries representing different geographical areas (the Czech Republic, Spain, Sweden, Switzerland and United Kingdom) and by socio-demographic groups. Environmental attitudes were assessed using the validated Environmental Attitudes Inventory (EAI-24), which considers ‘preservationist’ attitudes (which measures the extent to which people perceive that preserving and maintaining ecosystems and the diversity of natural species is important), and ‘utilitarian’ attitudes (which measures the belief that using and altering ecosystems and natural species for human objectives is right, appropriate and necessary) [54–56]. Items which assess perceived environmental threats have validity given they have been drawn from a priori qualitative research [28]. The analysis has aimed to determine how general environmental attitudes relate to perceptions of specific environmental threats. The analysis also considers the relationship between utilitarian and preservationist attitudes, and perceptions of environmental threat. It is predicted that pro-environmental attitudes will be positively associated with greater levels of perceived environmental threats, and that this association will vary by age and between genders, education level, and between participants who are urban or rural dwellers.*

**Comment 2:**

2. It is better to have a separate research area section to support the explanation and show the research area map with latitude and longitude information.

**Authors’ response:**

Many thanks for this suggestion. We have added a paragraph to explain potential differences in citizens’ environmental attitudes and perceived threats to the countryside across countries with reference to variation in environmental conditions across biogeographical regions. This makes it crucial to investigate both similarities and differences across the focused countries to better inform future policy-making (**lines 227-237**). We also agree that a map would be useful. We have created a new map indicating the locations of the countries and the corresponding biogeographical zones we are focusing on, including latitude and longitude information (**Fig. 1**).

**lines 227-237**

*Sample recruitment and survey data collection were undertaken by a social research agency (Qualtrics LLC) across five countries (N=3190): the Czech Republic (n=649); Spain (n=623); Sweden (n=645); Switzerland (n=641); United Kingdom (UK) (n=632). These countries were selected to represent the Continental, Mediterranean, Boreal, Alpine and Atlantic biogeographical regions respectively (see Fig. 1). In Europe, the Alpine region (Switzerland) and the Boreal region (Sweden) have the best conservation status for habitats and native species, whereas the Atlantic region (the UK) and the Continental region (Czech Republic) have the worst conservation status [57]. The Mediterranean region (Spain) has the largest area of degraded forests, grasslands, scrub and heath. Variations in* *environmental conditions across regions may lead to differences in citizens’ environmental attitudes and perceived environmental threats to the countryside [58].*

**Fig. 1:**

**Fig. 1**. Map of biogeographical regions of Europe included in the study

***Note***: Data are sourced from “Biogeographical regions in Europe” by European Environment Agency, 2017, (<https://www.eea.europa.eu/legal/copyright>).

**Comment 3:**

3. Figure 1 in the manuscript can be optimized further to show more research content and improve aesthetics.

**Authors’ response:**

We have taken the reviewer’s point and have added more information to the figure (now **Fig. 2**) about the perceived threats to the countryside and the assumed associations that were to be tested. We have also improved the aesthetics of the figure.

**Fig. 2**

**Fig. 2:** Environmental attitudes (EAI-24), endogenous variables and perceived threat to the countryside (PTC) plan for analysis

**Comment 4:**

4. Why did the author(s) not build the regression model equation and explain it?

**Authors’ response:**

We thank the reviewer for asking this question. We decided to use a conceptual framework (**now Fig. 2**) instead of building the regression model equation for two main reasons. **First**, regression equations are used with observed measures.  The statistical models in this paper involve both observed and latent variables. **Second**, we believe using a conceptual framework can better accommodate the readers. Further, we avoided stating the mathematical basis of each procedure as these are available in the wider literature. References are now supplied in support of the various statistical models associated with each of the 4 different procedures used (e.g. see **EFA: lines 293-294; MIMIC: lines 327-329; WLSMV: lines 337-341; Multiple-groups: lines 364-366**). In particular, after adding more information to Fig. 2 as the reviewer suggested in Comment 3, it shows better the associations that have been tested between different factors/variables, which is easier to understand for different readers. Codes for all the analyses are available from the authors upon request.

***References***

**Exploratory factor analysis (EFA)**

Asparouhov, T., & Muthén, B. (2009). Exploratory structural equation modeling. Structural Equation Modeling, 16, 397– 438. doi:10.1080/10705510903008204

Yates, A. (1987). Multivariate exploratory data analysis: A perspective on exploratory factor analysis. Albany, NY: State University of New York Press.

Browne, M. W. (2001). An overview of analytic rotation in exploratory factor analysis. *Multivariate Behavioral Research*, 36, 111–150. doi:10.1207/s15327906mbr3601_05

Hattoria M, Zhanga, G and Preacher K. J. (2017) Multiple Local Solutions and Geomin Rotation. MULTIVARIATE BEHAVIORAL RESEARCH VOL. 52, NO. 6, 720-731 <https://doi.org/10.1080/00273171.2017.1361312>

**MIMIC**

Bollen KA, Long JS. Testing Structural Equation Models. Newbury Park: Sage Publications; 1993.

Jöreskog KG, Goldberger AS. Estimation of a model with multiple indicators and multiple causes of a single latent variable. *J Am Stat Assoc*. 1975;70(351):631–639

**WLSMV**

Muthén, B. (1984). A general structural equation model with dichotomous, ordered categorical, and continuous latent variable indicators. *Psychometrika*, 49, 115-132.

Li, CH. Confirmatory factor analysis with ordinal data: Comparing robust maximum likelihood and diagonally weighted least squares. *Behav Res* **48**, 936–949 (2016). <https://doi.org/10.3758/s13428-015-0619-7>

**Multiple-groups**

Byrne, Shavelson, & Muthén, 1989; Cheung & Rensvold, 1999; Vandenberg & Lance, 2000).

Vandenberg, R. J., & Lance, C. E. (2000). A review and synthesis of the measurement invariance literature: Suggestions, practices, and recommendations for organizational research. *Organizational Research Methods*, 3(1), 4–70.

Byrne, B. M., Shavelson, R. J., & Muthén, B. (1989). Testing for the equivalence of factor covariance and mean structures: The issue of partial measurement invariance. *Psychological Bulletin*, 105(3), 456.

EFA models and their estimation (From: Hattoria M, Zhanga, G and Preacher K. J. (2017)

Muthén, B.O. (1998-2004). Mplus Technical Appendices. Los Angeles, CA: Muthén & Muthén

**Comment 5:**

5. The analysis of the discussion section is not deep enough, so it is suggested that the author(s) further enrich this section to highlight research contributions. At the same time, it should highlight the differences between previous studies.

**Authors’ response:**

Many thanks for this suggestion. We have revisited the literature and added citations throughout the discussion to contextualise the results, compare and contrast with previous results and reinforce the conclusions. The comparisons with other countries included, for example, the relationship between two dimensions of environmental attitudes (i.e. preservationist and utilitarian attitudes) (**lines 498-506**), differences in environmental attitudes across socio-demographic groups (**lines 513-521; 541-551**), and differences in perceived environmental threat across socio-demographic groups (**lines 611-620**). The discussion has also been re-structured for flow.

**lines 498-506**

*Consistent with previous research conducted in Portugal [32] and Ireland [74], preservationist and utilitarian attitudes were negatively correlated in four out of the five countries* *included in the research. However, Swiss citizens differed in this respect and appeared to have a more balanced distribution of the two attitudinal factors, with some believing that nature and natural species can be well protected whilst at the same time being used for human purposes. This is in keeping with existing research indicating the importance of societal demand to ecosystem management in Switzerland [75] and may be related to the fact that the Alpine region, where Switzerland is located, has the best conservation status of habitats in Europe [57].*

**lines 513-521**

*As expected, given previous research results [27,28], environmental attitudes varied demographically within and between countries. Differences in utilitarian attitudes between demographic groups were unexpected. Whereas utilitarian attitudes to the environment were more common among men in the UK, utilitarian attitudes were more prevalent among women in the Czech Republic, Spain and Switzerland. Existing research in contrast, has been consistent in the reporting that women tend to be more concerned about the environment [76,77] and that pro-environmental preservationist attitudes were more common among women [78]. Further research is required to understand these gender differences and how they relate to how the environment is used in these countries.*

**lines 541-551**

*Consistent with existing evidence for more positive environmental attitudes among those of higher socio-economic status [31], preservationist attitudes were more frequently observed among more educated respondents, but only in the UK. Respondents in the different countries also varied in the distribution of utilitarian attitudes with education level. As expected, given evidence for more positive environmental attitudes among those of higher socio-economic status (SES) [31], utilitarian attitudes were associated with having a lower education level in Spain. Contrary to our hypotheses, however, utilitarian attitudes were associated with a higher education level in the UK and Switzerland. This could reflect the large differences between countries in how people use the countryside and the degree to which people reported using the countryside for recreation and leisure (Czech Republic 49%; Spain 74%; Sweden 58%; Switzerland 63%; UK 79%).*

**lines 611-620**

*Although there were no demographic differences in perceived threat to the countryside in Switzerland and the UK, differences were observed between demographic groups in the other countries. Greater perceived threat to the countryside was higher among men in Sweden, increased with advancing age in Sweden and Spain, and with education level in Czech Republic. This finding contrasts with previous research indicating greater environmental awareness among younger people [34,92] or where no association with age was reported [93]. Demographic variation in perceived threat could reflect the relative salience of preservation and land use challenges faced in different regions and national variation in regulatory frameworks associated with recreation and agriculture [8]. Demographic differences may also vary between and within countries which differ in terms of their geographies and agri-ecology.*

**Comment 6:**

6. The author(s) should improve the timeliness of references to the manuscript and cite more high-quality literature in recent years.

**Authors’ response:**

The reviewer is correct in that relevant research has been published since the manuscript was compiled. Further material has been added throughout the introduction and discussion sections.

**Comment 7:**

7. It is suggested that the author(s) revise the manuscript to improve the quality of the languages and readability with the help of a native English speaker.

**Authors’ response:**

The authors thank the reviewer for their suggestion and have checked and adjusted the language and clarity of reporting throughout the manuscript. Parts of the introduction and discussion sections have been re-structured to improve readability.

**Response to Reviewer 2:**

**Comment 1:**

Reviewer #2: The study discusses the environmental attitudes of citizens in five countries (Czechia, Spain, Sweden, Switzerland and the United Kingdom) in relation to perceived threats and used the Environmental Attitude Inventory (EAI-24) method to measure citizens' environmental attitudes. The selection of topics makes sense and falls within the scope of the journal, but the following suggestions exist and require further reflection:

1. Data
The data combines questionnaires from five countries, and it is good that gender, place of residence, education level, etc., are combined in the demographic data. However, whether the proportion of women living in urban or rural areas is also 50 percent (as it is for men) requires further explanation. This should be included in the quota.

**Authors’ response:**

This is an important point and it has now been clarified in the method that the samples were quoted to be 50% urban/rural as well as 50% by gender (**lines 238-240**).

The null findings relating to rural/urban citizens have now been stated more clearly in the discussion. It has also been added that this was contrary to prediction given previous research which unlike our study, previous research has not employed large, cross-national, representative samples that renders our findings more reliable (**lines 561-566**).

**lines 238-240**

*Participants were quota sampled to be representative of national populations on age, gender (50%), socio-economic status, education level and to be 50% on rural versus urban residency.*

**lines 561-566**

*Neither preservationist nor utilitarian attitudes differed between rural and urban dwellers in any of the five countries. This was unexpected given previous evidence for more pro-environmental attitudes among rural dwellers [84] and evidence regarding polarised views on the prioritisation of resources in the countryside between rural and urban dwellers [28]. This may reflect the use of validated measures on large representative samples rendering the findings reliable at scale.*

**Comment 2:**

2. Framework
I see that the article puts a very simple framework at the end, and would suggest that this framework be added to the second part, and that a paragraph be added to describe the research design, methodology, and structure of the article.

**Authors’ response:**

Many thanks for this suggestion. A sentence has now been included toward the start of the method section, describing the framework, design and method (**lines 220-222**). We have also adapted our framework figure which now shows more information (now **Fig. 2**). In addition, we have now included a brief sentence on article structure toward the end of the introduction (**lines 205-217**).

**lines 220-222**

*The research compares environmental attitudes and perceived threat to the countryside cross-sectionally and the relationships therein across five European countries and in different demographics.*

**Fig. 2**

**Fig. 2:** Environmental attitudes (EAI-24), endogenous variables and perceived threat to the countryside (PTC) plan for analysis

**lines 205-217**

*Environmental attitudes were assessed using the validated Environmental Attitudes Inventory (EAI-24), which considers ‘preservationist’ attitudes (which measures the extent to which people perceive that preserving and maintaining ecosystems and the diversity of natural species is important), and ‘utilitarian’ attitudes (which measures the belief that using and altering ecosystems and natural species for human objectives is right, appropriate and necessary) [54–56]. Items which assess perceived environmental threats have validity given they have been drawn from a priori qualitative research [28]. The analysis has aimed to determine how general environmental attitudes relate to perceptions of specific environmental threats. The analysis also considers the relationship between utilitarian and preservationist attitudes, and perceptions of environmental threat. It is predicted that pro-environmental attitudes will be positively associated with greater levels of perceived environmental threats, and that this association will vary by age and between genders, education level, and between participants who are urban or rural dwellers.*

**Comment 3:**

3. Analysis and Discussion
The article starts with citizens with different environmental attitudes and compares the different outcomes of the five countries, but the analysis of the driving mechanism behind that outcome is insufficient, and further thought could be given to this aspect, and the discussion section needs to be further elevated to the height of the article. The advantages of having data across five countries are not fully realized, e.g., what are the influencing factors behind a given data result? Is the phenomenon specific to a particular country and why? How do different national circumstances (stage of development, economic structure, national spirit, policy trends, etc.) in different countries manifest themselves in the data results? The paper mentions the terrain represented by the different countries, Continental, Mediterranean, Boreal, Alpine, Atlantic, but there is no discussion of these factors in the analysis, and whether the citizens of the different countries have their environmental attitudes connected to the place.

**Authors’ response:**

We thank the reviewer for this comment and have added to the discussion section that Swiss citizens appeared to have a more balanced distribution of the two attitudinal factors, with some believing that nature and natural species can be well protected whilst at the same time as being used for human purposes. However, for other countries, preservationist and utilitarian attitudes appeared to be mutually exclusive. We discussed that it may be related to the fact that the Alpine region, where Switzerland is located, has the best conservation status of habitats in Europe. This may have made Swiss citizens more likely to believe they can balance the use and protection of nature (**lines 498-507)**.

We have also noted that perceived threat to the countryside was greatest in the UK, including all the activities related to environmental degradation. On the one hand, we attributed this partly to the Atlantic region, where the UK is located, having the worst conservation status of habitats among the five biogeographical regions included. This poor status might have led to stronger public concerns about environmental issues. On the other hand, we have also discussed potential regulatory and political factors, such as BREXIT, which has caused uncertainties in relevant policies and heightened public concerns (**lines 579-588**).

The cross-country comparative aspects have also been further developed with reference to recent policy review and stakeholder analysis conducted within these regions (Hunter et al., 2023, i.e. reference [88]) (**lines 588-593; 594-599**).

**lines 498-507**

*Consistent with previous research conducted in Portugal [32] and Ireland [74], preservationist and utilitarian attitudes were negatively correlated in four out of the five countries included in the research. However, Swiss citizens differed in this respect and appeared to have a more balanced distribution of the two attitudinal factors, with some believing that nature and natural species can be well protected whilst at the same time being used for human purposes. This is in keeping with existing research indicating the importance of societal demand to ecosystem management in Switzerland [75] and may be related to the fact that the Alpine region, where Switzerland is located, has the best conservation status of habitats in Europe [57]. However, for the other countries,* *preservationist and utilitarian attitudes appeared to be mutually exclusive.*

**lines 579-588**

*Perceived threats to the countryside were rated as greatest in the UK where nine out of the fifteen threats were rated higher than any of the other countries, including all the activities related to environmental degradation. This may be partly due to the fact that, of the five biogeographical regions included, the Atlantic region, where the UK is located, has the worst conservation status of habitats [57]. This poor status might have led to stronger public concerns about environmental issues. Levels of perceived environmental threat associated with food production have also been found to be higher in the UK compared to China [46]. This could also be attributed to land-use, regulatory and political factors specific to the region, for example, BREXIT [88] and attitudes towards related governance and national policies.*

**lines 588-593**

*Spain scored highest on four perceived threats related to improper land use and socio-economic uncertainty. This reflects the outcomes of in a recent consultation with stakeholders on countryside management which highlighted extensive perceived policy weakness in Spain [88]. Threat perceptions could also have been associated with socio-economic issues specific to the Spanish region such as those related to coastal development and over development of the -tourism [89].*

**lines 594-599**

*Perceived threats to the countryside that were associated with specific issues also varied across countries. For instance, highest perceived threat was associated with “bad behaviour of visitors” in the Czech Republic, Switzerland and the UK, and with “lack of young farmers taking over farming” in Spain and Sweden. Concern for the sustainability of farming has been reflected in a previous policy analysis [88] with stakeholder in Spain and Sweden arguing a need for policies to incentivise farming and to encourage more sustainable farming practices.*

**Comment 4:**

4. Significance
The significance of the study is somewhat general and requires further thinking. The factors that contribute to the different situations in different countries are organic and integrated, and conclusions simply attributed to media campaigns and policies are insufficient and not specific enough.

**Authors’ response:**

We have taken the reviewer’s point and. We have now cited further research throughout the manuscript, including recent studies in support for the study rationale for the study (Introduction) and comparing the results with those of other studies and in evidence for the intervention and policy recommendations (Discussion). Both the Introduction and Discussion sections have been re-structured for flow and readability. At the same time, we have been mindful that we cannot go too far beyond our data in interpreting our results.

**Comment 5:**

What efforts have countries with higher citizen environmental attitudes made? What lessons can be learned and improved?

**Authors’ response:**

Many thanks for this comment. In response to this, comment, we have briefly discussed why Swiss citizen had lowest preservation attitude and highest utilitarian attitudes among the countries included in our study (**lines 496-512**). In addition, we have been cautious in keeping any conclusions within the scope of our data and that of previous research studies, including a recently published policy review and stakeholder analysis conducted within these regions (Hunter et al., 2023, i.e. reference [88]) (**lines 588-593; 594-599**).

**lines 496-512**

*Preservationist attitudes were more prevalent in the sample compared to utilitarian attitudes in all five countries. Although the proportion of preservationist and utilitarian attitudes differed between countries, effect sizes were small. Consistent with previous research conducted in Portugal [32] and Ireland [74], preservationist and utilitarian attitudes were negatively correlated in four out of the five countries included in the research. However, Swiss citizens differed in this respect and appeared to have a more balanced distribution of the two attitudinal factors, with some believing that nature and natural species can be well protected whilst at the same time being used for human purposes. This is in keeping with existing research indicating the importance of societal demand to ecosystem management in Switzerland [75] and may be related to the fact that the Alpine region, where Switzerland is located, has the best conservation status of habitats in Europe [57]. However, for the other countries, preservationist and utilitarian attitudes appeared to be mutually exclusive. The observation that preservationist attitudes were lowest, and utilitarian attitudes highest, in Switzerland, implies a need for specific policies and communications to encourage people in Switzerland to shift towards more pro-environmental attitudes, potentially focused on the multi-functional nature of landscapes and the services which they deliver, accounting for the regulation and maintenance ES.*

**lines 588-593**

*Spain scored highest on four perceived threats related to improper land use and socio-economic uncertainty. This reflects the outcomes of in a recent consultation with stakeholders on countryside management which highlighted extensive perceived policy weakness in Spain [88]. Threat perceptions could also have been associated with socio-economic issues specific to the Spanish region such as those related to coastal development and over development of the -tourism [89].*

**lines 594-599**

*Perceived threats to the countryside that were associated with specific issues also varied across countries. For instance, highest perceived threat was associated with “bad behaviour of visitors” in the Czech Republic, Switzerland and the UK, and with “lack of young farmers taking over farming” in Spain and Sweden. Concern for the sustainability of farming has been reflected in a previous policy analysis [88] with stakeholder in Spain and Sweden arguing a need for policies to incentivise farming and to encourage more sustainable farming practices.*
