# Supplementary material for: Citizen attitudes towards the environment and association with perceived threats to the countryside: Evidence from countries in five European biogeographic zones
Source: PLoS One. 2024 Oct 10;19(10):e0311056. doi: 10.1371/journal.pone.0311056 (PMC11469601; doi:10.1371/journal.pone.0311056)
Supplement: Attachment — Submitted filename: Response to Reviewers.docx [file pone.0311056.s004.docx]

**Authors’ Response to Reviewer 2**

**Response to the second-round comments:**

Reviewer #2: The revisions to the article have improved considerably this time and basically meet the requirements for publication, with the following additional comments provided for reference

**Authors’ response:**

We appreciate the reviewer’s second-round comments and have addressed them in detail below. In addition, the reviewer noted that they did not receive our response to the first-round comments, even though we had included all responses when we resubmitted the manuscript and relevant documents. Therefore, we have attached the previous response to this document, with updated line numbers and fine-tuned content in accordance with the latest manuscript.

**Comment 1:**
1. Please revise Figure 2 to increase the readability of the image.

**Authors’ response:**

Following the reviewer’s advice, we have further revised Figure 2, simplifying the version we created after addressing the first-round comments. Our main goal is to present a clear framework that effectively highlights the categories and detailed factors included. We have also added an explanation of the correlations to the end of the figure title.

**Fig. 2**

**Fig. 2:** Environmental attitudes (EAI-24), endogenous variables and perceived threat to the countryside (PTC) plan for analysis. Arrow A represents the assumed association between environmental attitudes and perceived threat to the countryside; Arrow B represents the assumed association between socio-demographics and environmental attitudes; Arrow C represents the assumed association between socio-demographics and perceived threat to the countryside.

**Comment 2:**

2. check the language of the article

**Authors’ response:**

In the first-round revision, the language of our manuscript was improved by some of our co-authors who are native English speakers. In this second-round revision, we have further refined the language.

**Comment 3:**
3. why don't I see the author's responses to the reviewers?

**Authors’ response:**

We uploaded all the responses to all the reviewers during the first-round revision. In case the reviewer did not received the document, we have attached the previous response to this current document, with updated line numbers and fine-tuned content in accordance with the latest manuscript.

**Response to Reviewer 2 in the first-round revision:**

**Comment 1:**

Reviewer #2: The study discusses the environmental attitudes of citizens in five countries (Czechia, Spain, Sweden, Switzerland and the United Kingdom) in relation to perceived threats and used the Environmental Attitude Inventory (EAI-24) method to measure citizens' environmental attitudes. The selection of topics makes sense and falls within the scope of the journal, but the following suggestions exist and require further reflection:

1. Data
The data combines questionnaires from five countries, and it is good that gender, place of residence, education level, etc., are combined in the demographic data. However, whether the proportion of women living in urban or rural areas is also 50 percent (as it is for men) requires further explanation. This should be included in the quota.

**Authors’ response:**

This is an important point and it has now been clarified in the method that the samples were quoted to be 50% urban/rural as well as 50% by gender (**lines 238-240**).

The null findings relating to rural/urban citizens have now been stated more clearly in the discussion. It has also been added that this was contrary to prediction given previous research which unlike our study, previous research has not employed large, cross-national, representative samples that renders our findings more reliable (**lines 550-555**).

**lines 238-240**

*Participants were quota sampled to be representative of national populations on age, gender (50%), socio-economic status, education level and to be 50% on rural versus urban residency.*

**lines 550-555**

*Neither preservationist nor utilitarian attitudes differed between rural and urban dwellers in any of the five countries. This was unexpected given previous evidence for more pro-environmental attitudes among rural dwellers [84] and evidence regarding polarised views on the prioritisation of resources in the countryside between rural and urban dwellers [28]. This may reflect the present study’s use of validated measures on large representative samples rendering the findings reliable at scale.*

**Comment 2:**

2. Framework
I see that the article puts a very simple framework at the end, and would suggest that this framework be added to the second part, and that a paragraph be added to describe the research design, methodology, and structure of the article.

**Authors’ response:**

Many thanks for this suggestion. A sentence has now been included toward the start of the method section, describing the framework, design and method (**lines 220-222**). We have also adapted our framework figure which now shows more information (now **Fig. 2**). In addition, we have now included a brief sentence on article structure toward the end of the introduction (**lines 206-217**).

**lines 220-222**

*The research compares environmental attitudes and perceived threat to the countryside cross-sectionally and the relationships therein across five European countries and in different demographics.*

**Fig. 2**

**Fig. 2:** Environmental attitudes (EAI-24), endogenous variables and perceived threat to the countryside (PTC) plan for analysis. Arrow A represents the assumed association between environmental attitudes and perceived threat to the countryside; Arrow B represents the assumed association between socio-demographics and environmental attitudes; Arrow C represents the assumed association between socio-demographics and perceived threat to the countryside.

**lines 206-217**

*Environmental attitudes have been assessed using the validated Environmental Attitudes Inventory (EAI-24), which considers ‘preservationist’ attitudes (the extent to which people perceive that preserving and maintaining ecosystems and the diversity of natural species is important), and ‘utilitarian’ attitudes (the belief that using and altering ecosystems and natural species for human objectives is right, appropriate and necessary) [54–56]. Items which have assessed perceived environmental threats have validity given they have been drawn from a priori qualitative research [28]. The analysis has sought to determine how utilitarian and preservationist environmental attitudes relate to perceptions of specific environmental threats. It is predicted that pro-environmental attitudes will be positively associated with greater levels of perceived environmental threats, and that this association will vary by age and between genders, education level, and between urban and rural dwellers.*

**Comment 3:**

3. Analysis and Discussion
The article starts with citizens with different environmental attitudes and compares the different outcomes of the five countries, but the analysis of the driving mechanism behind that outcome is insufficient, and further thought could be given to this aspect, and the discussion section needs to be further elevated to the height of the article. The advantages of having data across five countries are not fully realized, e.g., what are the influencing factors behind a given data result? Is the phenomenon specific to a particular country and why? How do different national circumstances (stage of development, economic structure, national spirit, policy trends, etc.) in different countries manifest themselves in the data results? The paper mentions the terrain represented by the different countries, Continental, Mediterranean, Boreal, Alpine, Atlantic, but there is no discussion of these factors in the analysis, and whether the citizens of the different countries have their environmental attitudes connected to the place.

**Authors’ response:**

We thank the reviewer for this comment and have added to the discussion section that Swiss citizens appeared to have a more balanced distribution of the two attitudinal factors, with some believing that nature and natural species can be well protected whilst at the same time as being used for human purposes. However, for other countries, preservationist and utilitarian attitudes appeared to be mutually exclusive. We discussed that it may be related to the fact that the Alpine region, where Switzerland is located, has the best conservation status of habitats in Europe. This may have made Swiss citizens more likely to believe they can balance the use and protection of nature (**lines 488-497)**.

We have also noted that perceived threat to the countryside was greatest in the UK, including all the activities related to environmental degradation. On the one hand, we attributed this partly to the Atlantic region, where the UK is located, having the worst conservation status of habitats among the five biogeographical regions included. This poor status might have led to stronger public concerns about environmental issues. On the other hand, we have also discussed potential regulatory and political factors, such as BREXIT, which has caused uncertainties in relevant policies and heightened public concerns (**lines 569-577**).

The cross-country comparative aspects have also been further developed with reference to recent policy review and stakeholder analysis conducted within these regions (Hunter et al., 2023, i.e. reference [88]) (**lines 577-582; 583-588**).

**lines 488-497**

*Consistent with previous research conducted in Portugal [32] and Ireland [74], preservationist and utilitarian attitudes were negatively correlated in four out of the five countries* *included in the research. Swiss citizens differed in this respect and appeared to have a more balanced distribution of the two attitudinal factors, with some believing that nature and natural species can be well protected whilst at the same time being used for human purposes. This is in keeping with existing research indicating the importance of societal demand to ecosystem management in Switzerland [75] and that the Alpine region, where Switzerland is located, is considered to have the best conservation status of all habitats in Europe [57]. This contrasts with the other countries, where preservationist and utilitarian attitudes appeared to be mutually exclusive.*

**lines 569-5****77**

*Perceived threats to the countryside were rated as greatest in the UK. Nine out of the fifteen threats, including all those related to environmental degradation, were rated higher in the UK than in any of the other countries. Of the five biogeographical regions included, the Atlantic region, where the UK is located, has the worst conservation status of habitats [57]. This poor status might have led to stronger public concerns about environmental issues. Levels of perceived environmental threat associated with food production have also been found to be higher in the UK compared to China [46]. This could also be attributed to land-use, regulatory and political factors specific to the region, for example, BREXIT [88] and attitudes towards related governance and national policies.*

**lines 577-582**

*Spain scored highest on four perceived threats related to improper land use and socio-economic uncertainty. This reflects the outcomes of a recent consultation with stakeholders on countryside management which highlighted extensive perceived policy weaknesses in Spain [88]. Threat perceptions could also have been associated with socio-economic issues specific to the Spanish region such as those related to coastal development and over-tourism [89].*

**lines 583-588**

*Perceived threats to the countryside that were associated with specific issues also varied across countries. For instance, the highest perceived threat was “bad behaviour of visitors” in the Czech Republic, Switzerland and the UK, and “lack of young farmers taking over farming” in Spain and Sweden. Concern for the sustainability of farming has also been indicated in a previous policy analysis [88] with stakeholders in Spain and Sweden arguing a need for policies to incentivise farming and to encourage more sustainable farming practices.*

**Comment 4:**

4. Significance
The significance of the study is somewhat general and requires further thinking. The factors that contribute to the different situations in different countries are organic and integrated, and conclusions simply attributed to media campaigns and policies are insufficient and not specific enough.

**Authors’ response:**

We have taken the reviewer’s point and. We have now cited further research throughout the manuscript, including recent studies in support for the study rationale for the study (Introduction) and comparing the results with those of other studies and in evidence for the intervention and policy recommendations (Discussion). Both the Introduction and Discussion sections have been re-structured for flow and readability. At the same time, we have been mindful that we cannot go too far beyond our data in interpreting our results.

**Comment 5:**

What efforts have countries with higher citizen environmental attitudes made? What lessons can be learned and improved?

**Authors’ response:**

Many thanks for this comment. In response to this, comment, we have briefly discussed why Swiss citizen had lowest preservation attitude and highest utilitarian attitudes among the countries included in our study (**lines 486-501**). In addition, we have been cautious in keeping any conclusions within the scope of our data and that of previous research studies, including a recently published policy review and stakeholder analysis conducted within these regions (Hunter et al., 2023, i.e. reference [88]) (**lines 577-582; 583-588**).

**lines 486-501**

*Preservationist attitudes were more prevalent than utilitarian attitudes in all five countries. Although the proportion of preservationist and utilitarian attitudes differed between countries, effect sizes were small. Consistent with previous research conducted in Portugal [32] and Ireland [74], preservationist and utilitarian attitudes were negatively correlated in four out of the five countries included in the research. Swiss citizens differed in this respect and appeared to have a more balanced distribution of the two attitudinal factors, with some believing that nature and natural species can be well protected whilst at the same time being used for human purposes. This is in keeping with existing research indicating the importance of societal demand to ecosystem management in Switzerland [75] and that the Alpine region, where Switzerland is located, is considered to have the best conservation status of all habitats in Europe [57]. This contrasts with the other countries,* *where preservationist and utilitarian attitudes appeared to be mutually exclusive. The observation that preservationist attitudes were lowest, and utilitarian attitudes highest, in Switzerland, implies a need for specific policies and communications to encourage people in Switzerland to shift towards more pro-environmental attitudes, potentially focused on the multi-functional nature of landscapes and the services which they deliver, accounting for the regulation and maintenance ES.*

**lines 577-582**

*Spain scored highest on four perceived threats related to improper land use and socio-economic uncertainty. This reflects the outcomes of a recent consultation with stakeholders on countryside management which highlighted extensive perceived policy weaknesses in Spain [88]. Threat perceptions could also have been associated with socio-economic issues specific to the Spanish region such as those related to coastal development and over-tourism [89].*

**lines 583-588**

*Perceived threats to the countryside that were associated with specific issues also varied across countries. For instance, the highest perceived threat was “bad behaviour of visitors” in the Czech Republic, Switzerland and the UK, and “lack of young farmers taking over farming” in Spain and Sweden. Concern for the sustainability of farming has also been indicated in a previous policy analysis [88] with stakeholders in Spain and Sweden arguing a need for policies to incentivise farming and to encourage more sustainable farming practices.*
